# Rational Vaccine Design in Times of Emerging Diseases: The Critical Choices of Immunological Correlates of Protection, Vaccine Antigen and Immunomodulation

**DOI:** 10.3390/pharmaceutics13040501

**Published:** 2021-04-06

**Authors:** Virgil Schijns, Dragomira Majhen, Peter van der Ley, Aneesh Thakur, Artur Summerfield, Rita Berisio, Cristina Nativi, Alberto Fernández-Tejada, Carmen Alvarez-Dominguez, Sveinbjörn Gizurarson, Alla Zamyatina, Antonio Molinaro, Camillo Rosano, Žiga Jakopin, Ihsan Gursel, Siobhán McClean

**Affiliations:** 1 Intravacc, Institute for Translational Vaccinology (Intravacc), Utrecht Science Park, 3721 MA Bilthoven, The Netherlands; peter.van.der.ley@intravacc.nl; 2 Epitopoietic Research Corporation (ERC), 5374 RE Schaijk, The Netherlands; 3 Laboratory for Cell Biology and Signalling, Division of Molecular Biology, Ruđer Bošković Instiute, HR-10000 Zagreb, Croatia; Dragomira.Majhen@irb.hr; 4 Department of Pharmacy, University of Copenhagen, 2100 Copenhagen, Denmark; aneesh.thakur@sund.ku.dk; 5 Institute of Virology and Immunology, 3147 Mittelhausern, Switzerland; artur.summerfield@vetsuisse.unibe.ch; 6 Department of Infectious Diseases and Pathobiology, Vetsuisse Faculty, University of Bern, 3001 Bern, Switzerland; 7 Institute of Biostructures and Bioimaging, National Research Council, I-80134 Naples, Italy; rita.berisio@cnr.it; 8 Department of Chemistry “Ugo Schiff”, University of Florence, 50019 Sesto Fiorentino, Italy; cristina.nativi@unifi.it; 9 Chemical Immunology Laboratory, Center for Cooperative Research in Biosciences (CIC bioGUNE), Biscay Science and Technology Park, 48160 Derio-Bilbao, Spain; afernandeztejada@cicbiogune.es; 10 Ikerbasque, Basque Foundation for Science, 48009 Bilbao, Spain; 11 Facultativo en plantilla (Research Faculty), Instituto de Investigación Marqués de Valdecilla (IDIVAL), 39011 Santander, Spain; carmen.alvarezd@scsalud.es; 12 Faculty of Pharmaceutical Sciences, University of Iceland, 107 Reykjavik, Iceland; sveinbj@hi.is; 13 Department of Pharmacy, College of Medicine, University of Malawi, Blantyre 3, Malawi; 14 Department of Chemistry, University of Natural Resources and Life Sciences, 1190 Vienna, Austria; alla.zamyatina@boku.ac.at; 15 Department of Chemical Sciences, University of Napoli Federico II, Complesso Universitario Monte Santangelo, I-80126 Napoli, Italy; molinaro@unina.it; 16 Department of Chemistry, School of Science, Osaka University, 1-1 Osaka University Machikaneyama, Toyonaka, Osaka 560-0043, Japan; 17 Proteomics and Mass Spectrometry Unit, IRCCS Policlinico San Martino, 16132 Genova-1, Italy; camillo.rosano@hsanmartino.it; 18 Faculty of Pharmacy, University of Ljubljana, 1000 Ljubiljana, Slovenia; ziga.jakopin@ffa.uni-lj.si; 19 Molecular Biology and Genetics Department, Science Faculty, Bilkent University, Bilkent, 06800 Ankara, Turkey; ihsangursel@bilkent.edu.tr; 20 School of Biomolecular and Biomedical Sciences, University College Dublin, Belfield, D04 V1W8 Dublin, Ireland

**Keywords:** rational, vaccine, design, antigen, adjuvant, delivery system, route, immunization

## Abstract

Vaccines are the most effective medical intervention due to their continual success in preventing infections and improving mortality worldwide. Early vaccines were developed empirically however, rational design of vaccines can allow us to optimise their efficacy, by tailoring the immune response. Establishing the immune correlates of protection greatly informs the rational design of vaccines. This facilitates the selection of the best vaccine antigens and the most appropriate vaccine adjuvant to generate optimal memory immune T cell and B cell responses. This review outlines the range of vaccine types that are currently authorised and those under development. We outline the optimal immunological correlates of protection that can be targeted. Finally we review approaches to rational antigen selection and rational vaccine adjuvant design. Harnessing current knowledge on protective immune responses in combination with critical vaccine components is imperative to the prevention of future life-threatening diseases.

## 1. Introduction

Vaccines have proven highly successful in reducing mortality and morbidity of infectious diseases on a global scale. The recent COVID-19 viral pandemic and the potential spread and threat of newly emerging infections demands for new efficient vaccines now and in the future [1]. An effective vaccine is designed to generate artificial adaptive immunity by instructing the immune system to either respond to future infections or to act therapeutically against an established disease or cancer. Ideally it is a medication which can be safely and conveniently administered to generate the most appropriate long-lasting prophylactic or therapeutic immunity [2]. In the last century successful vaccines have been generated empirically, in various formats with a trend to use more refined, safer antigens. Unfortunately, many of these trial-and-error vaccines still show limitations such as a limited efficacy, poor stability, and repeated dose requirements. Modern rational vaccine design involves certain crucial decision steps. Ideally, such strategic decisions involve targeting the immune response to make the vaccine effective, the choice of the antigen, its delivery and presentation, as well as the choice of immune response-inducing and shaping vaccine adjuvant or immunomodulator.

Here we describe the principal vaccine concepts ranging from live attenuated to peptide vaccines and their most common advantages and disadvantages. Importantly, depending on the aim of the vaccine its rational design first requires the identification of the immune correlate(s) of vaccine efficacy or the so-called immunological correlates of protection (IMCOP). Next, a suitable immunogenic vaccine antigen has to be identified, and subsequently formulated and presented in order to stimulate the adaptive immune system for the generation of memory immune T and/or B cells. We will address these important decision steps for different types of vaccines, which require a rational approach for the selection of the suitable antigen(s) with low variability, and a critical choice of co-formulated, or built-in (optimized), vaccine adjuvant as antigen delivery system and/or immunomodulator.

## 2. Vaccine Types for Artificial Adaptive Immunity

### 2.1. Live Attenuated Vaccines

Live attenuated vaccines (LAV) are widely considered highly effective vaccines, as they closely mimic natural immunity. Indeed, live vaccines are among the most successful vaccines as exemplified by the smallpox vaccine (live vaccinia virus) which led to smallpox eradication in 1980 and the poliomyelitis vaccine which led to polio being almost completely eradicated. Typically, live strains were attenuated by deletion of genes which impeded virulence or conferred auxotrophic phenotypes. LAVs are highly immunogenic, consequently do not require additional adjuvants. A single dose often confers protective immunity, but safety concerns can mean they are not suitable for immunocompromised individuals. For example, the Sabin polio vaccine strains showed issues with safety in a small cohort of recipients due to reversion to a paralytic phenotype resulting in vaccine-associated polio cases, with an incidence of one in 500,000 first time recipients. Attenuation mutations were lost in vaccine-related polio cases and multiple changes in known attenuation mutations were identified in virus excreted from vaccinated children [3]. This potential reversion to virulence highlights potential critical risk of live attenuated vaccines and the need for rational design of live attenuated vaccine strains.

Additional advantages such as stability on storage, avoiding the need for cold chain and associated costs and challenges can be engineered into LAVs. The Flumist^®^ vaccine is an LAV that incorporates all these characteristics [4]. The trivalent live attenuated seasonal vaccine consists of three live influenza viruses (two type A and one type B). Each virus is comprised of a genetic rearrangement containing six gene segments from master donor viruses. The gene segments encoding hemagglutinin and neuraminidase elicit the protective immune responses, while the other gene segments comprise the genetic backbone and confer temperature sensitive, cold adapted, and attenuated phenotypes. The LAV influenza vaccine is well tolerated in children and in healthy adults and has been reported to be superior to the trivalent inactivated influenza vaccine in terms of reduced attack rate and disease severity in recipients with breakthrough influenza [4]. Recently developed live attenuated viral vaccines are incorporated in recombinant vectors, for example the dengue vaccine first licensed in December 2015 [5]. This is a tetravalent recombinant LAV with a yellow fever 17D vaccine virus backbone. It has demonstrated a good safety and efficacy profile in clinical trials as described below (Section 2.5).

Live attenuated bacterial vaccines have also been developed and licensed, or are at various stages of development, including vaccine candidates for *Vibrio cholerae*, *Burkholderia pseudomallei*, *Salmonella typhimurium*, *Francisella tularensis* and *Pseudomonas aeruginosa*.

The typhoid live oral vaccine (Ty21a) is a licensed oral vaccine which was developed by chemical mutagenesis of a *Salmonella typhi* strain and incorporates an inactive galE gene encoding an enzyme involved in LPS core oligosaccharide biosynthesis and over 20 other mutations [6]. It generates potent mucosal, humoral and cellular immune responses and has a good safety record in large scale safety trials involving children and adults from Chile, Egypt and Indonesia. No vaccine-related adverse reactions or reversions were identified among 200 million recipients over 25 years [7]. Its safety and ability to induce robust T cell responses has led to its evaluation as an oral vaccine delivery platform for a range of alternative vaccines against shigellosis, anthrax, plague or HPV [8,9] (Section 2.6).

### 2.2. Inactivated “Killed” Vaccines

Inactivated or killed vaccines consist of preparations of isolated and amplified disease-inducing microorganisms which are subsequently made incompetent for replication by various inactivation methods, including heat-inactivation, chemical inactivation or radiation. Usually, these vaccine induce less strong or less lasting immunity when compared to replicating attenuated vaccines. Therefore, they often require booster immunizations and co-administered immunomodulatory adjuvants. Successful licensed inactivated vaccines have been developed against polio, rabies, hepatitis A and influenza. For the pandemic SARS-CoV2 virus inactivated killed viral vaccines are being developed, either as whole inactivated viral variants or as adjuvanted subunit (protein or peptide) vaccines.

### 2.3. Subunit Protein and Peptide Protein and Polysaccharide Vaccines

Subunit antigen vaccines contain specific essential antigens to elicit protective responses without any live components and consequently are considered very safe. However, they are generally of lower immunogenicity and thus require additional adjuvant(s) to promote their protective responses. Early subunit vaccines exploited the available knowledge on microbial pathogenesis, for example the diphtheria toxoid and tetanus toxoid antigens, which were prepared by formalin inactivation of the respective bacterial toxins. Other subunit vaccines are based on purified recombinant proteins, such as the hepatitis B surface antigen with an aluminum-based adjuvant in the licensed Hepatitis B vaccine [10] and the herpes zoster vaccine consisting of a single recombinant glycoprotein E with the AS01 adjuvant [11]. Vaccines based on polysaccharide antigens were introduced in the 1970s and while they were also safe and well tolerated, they are not immunogenic in children under two years; have relatively short acting immunity (3 to 5 years) and induces poor immunological memory [12]. Covalent conjugation of the polysaccharide components to carrier proteins greatly enhanced their immunogenicity and efficacy. The quadrivalent *Neisseria meningitides* vaccine MenACWY-CRM (Menveo^®^) and the MenACWY-TT (Nimenrix^®^) vaccines comprise purified polysaccharide from four major capsular sub-groups A, C, W and Y conjugated to either diphtheria cross reactive material (CRM) or tetanus toxoid (TT), respectively, are approved for use in infants of 6 weeks and older. The flexibility of the conjugate approach is exemplified by the development of pneumococcal conjugate vaccines (PCV) to protect against *Streptococcus pneumoniae*. The first PCV was licensed in 2000 and comprised of polysaccharide representing seven serotypes. It was subsequently replaced by versions with broader coverage, namely ten-valent (PCV10) and 13-valent (PCV13) versions.

Several strategies have been devised to identify novel vaccine antigens. Rappuoli and colleagues pioneered the use of reverse vaccinology to identify novel antigens against *Neisseria meningitides* serogroup B [13,14] as addressed in Section 4.1. This approach involved mining the sequenced genome to identify any surface-expressed proteins based on the presence of signal peptides, and subsequently evaluating the identified antigens for immunogenicity in mice. This predictive approach led to the development of the Men B vaccine, Bexero^®^ licensed in 2013 which has had a significant impact on meningococcal disease caused by serogroup B. While this approach has revolutionized vaccine antigen discovery, one key limitation relates to the costs associated with testing the vast array of surface proteins identified (e.g., 350 antigens in the case of meningococcal B (MenB)), all of which were tested in mice. Alternative approaches exploit bacterial host interactions including identification of immunoreactive proteins via immunoproteomic approaches or identifying protein-protein interactions. The former approach typically involves probing two dimensional blots prepared from bacterial proteins with serum from infected patients or animals to identify immunoreactive proteins that stimulate serological antibody responses. One limitation of this method is that humoral responses may not be ultimately protective, and identification of potential subunit antigens based on stimulation of a serological response may not identify protective antigens [15]. More recently immunoproteomic studies incorporating antigen processing and presentation elements allowed identification of vaccine antigens that stimulate helper and cytotoxic effector immune responses in addition to humoral responses and consequently may show more effective immune protection [16]. This involves immunoprecipitation of viral infected cell lysates by affinity chromatography with specific anti-HLA mAbs, subsequent purification and mass spectrometry.

Identification of bacterial proteins involved in host cell attachment represents another approach to the identification of subunit vaccine antigens capable of stimulating both humoral and cellular responses. Bacterial membrane proteins are probed with human epithelial cells representative of the site of mucosal colonization with subsequent identification of bacterial protein involved in host cell attachment by mass spectroscopy, thereby focusing the field of potential efficacious antigens identified and reducing the burden of testing potentially unnecessary proteins that although present on the microbial surface are not involved in the direct host cell response [17]. This approach has led to the identification of *Burkholderia* antigens that are protective and stimulate both a serological response and also contain potent T cell epitopes [18].

Identification of the most effective epitopes in a subunit protein antigen can result in smaller (20 to 30 amino acid) peptide-based vaccines which can be synthesized in vitro. Consequently, they can be readily purified, are safer and may potentially trigger the desired immunological response. However, such peptide epitopes generally require conjugation to other peptide epitopes or biopolymer carriers in order to overcome their relatively small size and improve immunogenicity. In most cases adjuvant formulations are required. No human peptide prophylactic vaccine has been licensed to date.

### 2.4. RNA Vaccines

Vaccines based on in vitro-transcribed mRNA have the potential to transiently express the encoded protein in situ without the adverse effects of viral and DNA-based constructs. Two major types of RNA that are currently studied as vaccines are non-replicating mRNA and virally derived self-amplifying RNA. Both have in common a cap structure, 5′ and 3′ untranslated regions (UTRs), an open-reading frame (ORF), and a 3′ poly(A) tail, while self-amplifying RNA additionally contains replication machinery derived from positive-stranded mRNA viruses, most commonly from alphaviruses such as Sindbis and Semliki-Forest viruses [19]. In order to remove impurities, eliminate undesirable immune activation and improve translation, in vitro-transcribed mRNA purification is critical [20].

In order to be translated and elicit an antigen-specific immune response, an mRNA-vaccine has to reach the cytosol of its target cells. There are two basic approaches for the delivery of mRNA vaccines: (1) loading of mRNA into dendritic cells (DCs) ex vivo, followed by re-infusion of the transfected DCs, or (2) direct injection of mRNA with or without a carrier into target cells. The most common carriers used for complexing mRNA are protamine (a cationic peptide), lipid or polymer-based nanoparticles. In addition to facilitating efficient cell delivery, the complexation with different carriers may influence immunogenicity and longevity of the mRNA vaccine. As well as inducing specific immune response toward the encoded antigen, once in the endosome, mRNA is recognized by RNA-sensing receptors, namely Toll-like Receptors (TLRs), and other pattern recognition receptors (PRR) contributing to the immunostimulatory features of mRNA vaccine. Alternatively, an early shut-down of antigen expression after the mRNA vaccination due to unwanted PRRs activation might be detrimental underlining the need for fine tuning of mRNA recognition by host immune system.

In recent years an increasing number of preclinical studies have shown promising results with both self-amplifying and non-replicating mRNA vaccines conferring protection against various pathogens. However, data reporting outcomes of clinical trials are still modest. The first-ever demonstration in humans shows that a prophylactic mRNA-based candidate vaccine against rabies virus induced boostable functional antibodies against a viral antigen when administered intradermally with a needle-free device, although not when injected intramuscularly by a needle-syringe. The vaccine was generally safe with a reasonable tolerability profile [21]. By contrast, immunization with dendritic cells transfected with mRNA encoding HIV-1 Gag and Nef did not induce significant interferon-gamma producing enzyme-linked immunospot responses. However, proliferative responses to HIV-1 antigens and to a neo-antigen were increased, but the effects were transient. Thus, dendritic cell vaccination requires optimization to elicit stronger and long-lasting immune responses for this strategy to be effective as an HIV-1 therapeutic vaccine [22]. Nevertheless, encouraging data were obtained in a phase 1 dose-escalation, open-label trial to evaluate candidate vaccine mRNA-1273 encoding the stabilized prefusion SARS-CoV-2 spike protein. They revealed that this vaccine induced anti–SARS-CoV-2 immune responses in all participants, and no trial-limiting safety concerns were identified [23]. Two independent COVID-19 mRNA-vaccines were subsequently authorized by the FDA and EMA- for large-scale immunization campaigns against COVID-19 [24,25]. These recent data illustrate that RNA vaccines represent a modular platform technology for rapid development of vaccines against emerging disease as soon as the sequence of the target antigen is known.

### 2.5. DNA Vaccines

A DNA vaccine comprises the DNA that encodes vaccine antigens. After administration of the DNA to the host, antigens are produced in vivo and intended to stimulate an immune response. DNA vaccines contain nucleotides encoding an antigenic portion of a tumor-associated antigen or a target pathogen, such as the viral core region or viral envelope region. Any antigen encoded by a DNA vaccine is expressed intracellularly and consequently can be processed via the endogenous Major histocompatibility complex (MHC) class I pathway. Contrary to an RNA vaccine, protein expression by a DNA vaccine requires the nuclei acid to cross two cellular membranes, namely the plasma, as well as the nuclear membrane. Thus, successful transfection is crucial for DNA vaccine efficacy. Hence, various delivery methods have been developed, including gene gun, jet injection, in vivo electroporation, but also different formulations of DNA, for example encapsulation in lipid nanoparticles, adsorption to polymers, and adsorption or encapsulation in biodegradable nanoparticles. Safety concerns of DNA vaccines mostly relate to potential DNA integration into the host genome and generation of antibodies against the injected DNA. Indeed, DNA integration events were detected in a few studies, while no anti-DNA antibodies were found in animal models. In general, the potential risks of DNA vaccines are considered relatively low, although safety issues may differ from one application to another, and require strict monitoring [26]. To date, the DNA vaccine concept has been tested and applied against various pathogens and tumor antigens. In theory, this conceptually safe, non-replicating vaccine approach is a technically simple means of inducing immune responses. Importantly, DNA vaccines can induce both humoral and cellular immunity, which is the elusive aim of alternative vaccines [27].

Despite the initial studies on DNA vaccines commencing in the 1990s, there are no approved DNA vaccines for use in humans to date. There are, however, a selection of DNA-based vaccines approved by the USDA for veterinary use, including an equine vaccine against West Nile Virus [28] and canine melanoma vaccine [29]. One of the first human clinical trials with DNA vaccines evaluated the therapeutic and prophylactic effects against HIV and showed disappointing immunogenicity with poor T cell responses and low, or undetectable, humoral responses. [30]. More recently, DNA vectors have been used as a priming immunization in combination with protein [31], poxvirus [32] or adenovirus [33] as HIV vaccine in prime/boost regimens indicating that combination of vectors, may beneficially influence the quality of the immune responses. Evaluation of safety and immunogenicity for an anti-Middle East respiratory syndrome (MERS) coronavirus DNA vaccine showed that it was well tolerated with no serious adverse events. Immune responses proved dose-independent, detected in more than 85% of participants after two vaccinations [34]. Built on this prior experience a synthetic DNA-based vaccine candidate targeting the SARS-CoV-2 S protein, INO-4800, was generated. Preliminary studies conducted in mice and guinea pigs revealed that the INO-4800 vaccine induced both cellular and humoral host immune responses that were observed within days following a single immunization, including cross-reactive responses against SARS-CoV-2 virus. These data demonstrate the immunogenicity of this synthetic COVID-19 DNA vaccine candidate [35] supporting further evaluation of the DNA vaccine concept.

### 2.6. Recombinant Viral Vector Vaccines

Viruses have proven to be highly efficient vehicles for introducing foreign nucleic acid into target cells. Additionally, viruses are sensed by several intra- and extra-cellular Toll like receptors and intrinsically induce host immune responses upon cell infection. Both of these features, accompanied by extensive knowledge of molecular biology and methods for manipulating the viral genome that are now available, make viruses attractive candidates for vaccine vector development. Hence, recombinant viral vectors have been and are being investigated as vaccines targeting a broad range of viral, bacterial, and protozoan pathogens. They are particularly used in disease areas where classical vaccination strategies have proven ineffective, difficult, or technically impossible.

Viral genomes can be manipulated to express any antigen of choice encoded either in the genome [36] or presented as an epitope displayed on the surface of an unrelated, modified virus [37]. Delivery of the target antigen in the context of a viral vector allows faithful antigen generation and processing, namely correct protein folding, and modifications such as glycosylation. Once in the cell, viral vectors mimic the natural viral infection, thereby inducing potent immune responses. Hence, viral vector-based vaccines can be delivered without additional adjuvants while promoting strong antigen-specific cellular and humoral immune responses against the target antigen [38]. For the most commonly employed viral vector-based vaccines, high yield, and scalable production processes have been established [39]. Several viral vectors, including adenoviruses, parvoviruses, togaviruses, paramyxoviruses, rhabdoviruses, and poxviruses are currently being evaluated and developed as vaccine vectors [40].

Viral vectors present a versatile and modular platform for vaccine development that can be especially valuable in outbreak situation when prompt reaction is needed. The SARS-CoV-2 vector vaccine candidates, which we are currently witnessing with unprecedented development, exemplifies this. Viral vector-based vaccines against SARS-CoV-2 have been tested in the context of non-replicative vectors such as human and chimpanzee adenoviruses and replication-competent vesicular stomatitis virus [41]

The chimpanzee adenovirus-vectored vaccine ChAdOx1 nCoV-19, encoding the spike protein of SARS-CoV-2, was immunogenic in pigs and mice, eliciting a robust humoral and cell-mediated response [42]. Vaccination with ChAdOx1 nCoV-19 (both prime-only and prime-boost regimen) induced humoral and cellular immune response in rhesus macaques [43], showed an acceptable safety profile, and homologous boosting increased antibody responses [44]. Moreover, a recombinant adenovirus type-5-vectored COVID-19 vaccine was reported to be safe, tolerable and immunogenic inducing humoral and rapid specific T cell responses in healthy adults [45,46]. Induction of robust neutralizing antibody responses and complete or near-complete protection evidenced in bronchoalveolar lavage and nasal swabs following SARS-CoV-2 challenge in nonhuman primates was also shown for adenovirus type 26 vector-based vaccine for SARS-CoV-2, termed Ad26.COV2.S [47]. All three adenovirus-based SARS-CoV-2 vaccine candidates are in phase 3 clinical trials at time of writing and two are approved for emergency or limited use in the United Kingdom and China, respectively. Moreover, vaccination with replication-competent vesicular stomatitis virus (VSV) expressing a modified form of the SARS-CoV-2 spike generated neutralizing immune responses and protected mice from SARS-CoV-2 supporting development of VSV-SARS-CoV-2 as an attenuated, replication-competent vaccine against SARS-CoV-2 [41]. However, regardless many advantages and gathered knowledge, so far, few viral vector based vaccines, e.g., Dengvaxia, a recombinant Dengue vaccine based on the yellow fever attenuated strain 17D, have been licensed for human use [48].

### 2.7. Recombinant Bacterial Vector Vaccines

The most commonly used approaches to bacterial vaccines are based on inactivated whole cells, purified recombinant proteins and protein-polysaccharide conjugates. Genetically engineered live attenuated bacteria are currently not widely used as human vaccines, although this approach has been investigated especially in the case of the BCG vaccine against tuberculosis [49]. Outer Membrane Vesicles (OMVs) are gaining attention as novel vaccine candidates. OMVs contain bacterial surface components and virulence factors, including pathogen associated molecular patterns (PAMPs) that trigger innate immune responses, making them attractive as candidate vaccines. Most recently, the broad-coverage Men B OMV-based vaccine (Section 2.3) has proved to be effective in controlling meningococcal outbreaks [50,51]. The isolation of OMVs from *N meningitidis* requires treatment with deoxycholate detergent to in-crease the yield and to reduce endotoxin levels. Endotoxins are lipopolysaccharides (LPS) that are potent TLR-4 agonists inducing activation of innate immune responses and inflammatory cytokines secretion. Removal of LPS by detergent treatment is essential to prevent serious adverse effects, however this treatment leads to the removal of potential surface exposed antigens with consequent impact on the long-term stability of the OMV vaccine. Genetic modification of *N. meningitidis* by deletion of specific genes overcomes both these limitations. Deletion of the *rmpM* gene results in higher yields of OMV. The RmpM protein anchors the outer membrane to the peptidoglycan layer, and its absence consequently leads to increased OMV release [52]. The toxicity of OMVs can be attenuated by deletion of genes encoding the LpxL1 or LpxL2 enzymes of the lipid A biosynthesis pathway. This results in a penta-acylated form of LPS which has reduced endotoxic activity relative to hexa-acylated LPS [53]. The combined effect of these modifications enables the isolation of high yields of OMVs with reduced endotoxicity.

Vaccine applications of OMVs can be greatly expanded by the expression of heterologous antigens, where the OMV’s serve as both adjuvant and delivery vehicle. Surface exposure of the antigens might improve the immune response, so targeting to the OMV surface is an important consideration. Two different methods for surface display in *N. meningitidis* are available: (i) fusion to the N-terminus of the lipoprotein factor H binding protein followed by internal expression by the OMV-producing strain [54], and (ii) external linkage to OMVs of a separately produced recombinant protein carrying a C-terminal LPS-binding tag. The first method has the advantage that only a single vaccine preparation needs to be made, while the second method requires the combination of two separate products but allows greater flexibility in the choice of antigen. Genetic removal of immunodominant meningococcal surface antigens can be used to reduce the immune response against the OMV carrier itself.

## 3. The Immunological Correlate(s) of Protection

Before embarking on the design of a vaccine it is very important to first consider what type of immune response will generate the desired vaccine-induced immunity. While this may seem obvious, this approach was not followed for many existing vaccines developed for infectious diseases in the last century. As mentioned, many current vaccines have been developed empirically and the immunological correlates of protection are not yet clearly defined for many infectious diseases.

Historically most vaccines aimed to induce neutralizing antibody responses (see Section 3.2). Neutralizing antibodies are relatively easy to measure in the serum of immunized individuals and the detection of neutralizing antibodies in serum samples likely reflects their activity in the infected host. However, numerous infectious diseases and malignancies cannot be prevented or cured (in case of a therapeutic vaccine) by neutralizing antibodies. In most cases these infections show an intracellular life cycle, or a high degree of a variability of surface antigens, which cannot be targeted since they escape from vaccine-induced antibodies. Such diseases, if preventable at all, often require cell-mediated or cellular immunity (Section 3.2), preferably directed against a conserved non-variable antigen(s), to impede the disease. As a result, many vaccine approaches have failed due to their inadequate design and inability to induce a cell-mediated immune response.

### 3.1. Passive Immunity Transfer or Immunity Depletion

Knowledge of the contribution of specific immune effector mechanisms involved in protection against a particular pathogen or tumor, will help to identify the type of immune response that should be evoked by effectively-designed vaccines. In order to identify which type of immune reaction is necessary for protection against a particular disease a range of approaches can be followed. Animal models can be used to decipher the contribution of antibodies or specific immune cell populations. One strategy involves the assessment of passive protection of either antibodies or immune T cells from immune donors to naïve recipients that are subsequently exposed to an artificial challenge with the pathogen or tumor of interest. Another approach involves the selective depletion of either antibody-producing B cells or T cells (or their CD4 or CD8 subpopulations) in immune hosts and the subsequent monitoring of a drop in protective immune function after challenge. The contribution of antibody-producing B cells can be studied by using B cell-neutralizing antibodies or by specific genetic deletion of the B cell population (e.g., μMT gene deficient mice). Alternatively, the contribution of T cells to immunity in immune hosts can be examined using T cell-depleting antibodies, or mice with genetically deleted T cells (or their subpopulations). Any reduction in immunity relative to wild-type or depleted immune mouse models indicate their contribution.

Despite these experimental strategies, the specific contributions of distinct immune effector mechanisms are known for just a few pathogens. This may be due in part to the lack of suitable animal models reflecting human or veterinary disease, the complex changes in life cycle of certain pathogens and their evasion from immune surveillance, as well as the complex nature of immune reactions and synergistic activities of immune cell populations. For example, antibodies may be involved in protection during the acute phase of infection while cell-mediated responses may become critical during the chronic phase of infection. Although animal models may not fully reflect the disease or may not be available, in many cases there are clues to the signature of immune clearance mechanisms that are associated with recovery from, or immunity to, specific infectious diseases. Extrapolations to correlates of immunity can be made based on knowledge from related categories of intracellular versus extracellular pathogens, or related types of micro-organisms with similar life cycles. Actual correlates have been defined or suggested for Hib, pneumococcal and meningococcal vaccines. Moreover, for most tumors we know that in general type-1 immune and STING pathway interferon responses are related to protection [55]. Hence, the immune correlate of protection can be estimated or is predictable to some extend without the need for immunity transfer or depletion experiments.

The systems biology approach can facilitate a better picture of the immune responses in general and to vaccination in humans [56]; in fact, it may prove that vaccination-induced artificial immune effector clearance, rather than natural immune effector responses, may prove sufficient to fight of particular pathogens or tumors and may provide even better levels of protection. Understanding the immunological mechanisms of vaccination and the bridging of technical knowledge gaps [57] will help in the rational design of future vaccines against emerging infectious pathogens, such as COVID-19, as well as against prominent global diseases such as HIV, malaria, and tuberculosis.

### 3.2. Types of Protective Immune Responses

#### 3.2.1. Antibody-Based Immunity

**Figure 1:**
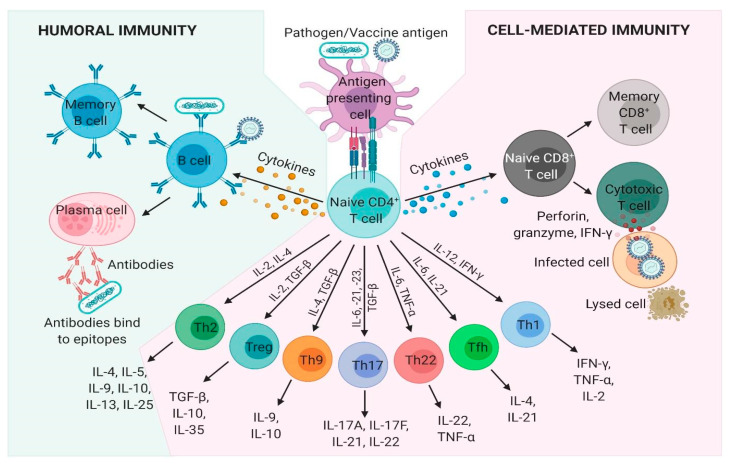
Humoral and cell-mediated adaptive immune responses. Adaptive immunity involves the activation of lymphocytes and develops following exposure to diseases or immunization against diseases through vaccination. Pathogen, tumor or vaccine antigens are recognized by antigen-presenting cells (APCs). Once processed, the antigenic peptides are presented on the surface of major histocompatibility complex (MHC)-II, which are recognized by T cell receptors (TCR) on naïve CD4^+^ T cells. Upon TCR activation by APCs, naïve CD4^+^ T cells differentiate into subpopulations: T helper (Th)1, Th2, Th9, Th17, Th22, T follicular helper (Tfh), and T regulatory cells (Treg) under unique cytokine-polarized environments. B-cells, following co-stimulation from cytokines produced by CD4^+^ T cells, transform into plasma cells that secrete antibodies which circulate in blood and extracellular fluid. A humoral immune response is mediated by secreted antibodies produced by B-cells, which are specific for an individual antigen. CD8^+^ T cells recognize and bind to intracellularly processed antigenic peptides through their TCR, which are presented on MHC-I molecules on the surface of APCs and infected cells. Cytokines released by CD4^+^ T cells also stimulate cytotoxic CD8^+^ T cells, which release effector molecules such as granzyme, perforin, and IFN-γ that destroy infected host cell. A subset of memory B and T cells confer future immunity to the cognate pathogen or antigen. IFN-γ, interferon gamma; IL, interleukin; TGF-β, transforming growth factor β; TNF-α, tumor necrosis factor α.

Humoral or antibody-based immunity is one of the two arms of the adaptive immune response, which results in the generation of antigen-specific antibodies that target invading microbial pathogens or vaccine antigens. Humoral immunity is achieved by B-cells but requires help from CD4^+^ T cells and therefore is also dependent on successful cell-mediated immunity. Activated B cells interact with antigen-specific CD4^+^ helper T cells in the outer cortex of the lymph nodes and undergo proliferation in the presence of cytokines such as IL-4 and IL-5 produced by CD4^+^ T cells. The antibodies produced bind the organisms and/or their toxins, directly interfering with microbial proliferation via neutralization, opsonization, and complement activation, or will direct other immune cells to phagocytose and destroy the bound microbe. Following a successful induction of humoral immune response, B cells producing affinity matured and isotype-switched antibodies differentiate into quiescent memory B cells (Figure 1).

#### 3.2.2. Cell-Mediated Immunity

Cell-mediated immunity is the other arm of the adaptive immune response that generates a wide variety of antigen specific effector T cells subsets which can either directly kill infected cells or induce various effector functions in conjunction with other immune cells. Naïve CD4^+^ T cells encounter pathogen or vaccine antigenic peptides complexed with MHC-II molecules that are presented on the surface of antigen presenting cells (APCs), such as dendritic cells and macrophages. Activated APCs provide the critical co-stimulatory signals, which stimulate the T cell receptor (TCR) resulting in T cell proliferation. Following TCR activation by APCs, naïve CD4^+^ T cells differentiate into either T helper (Th)1, Th2, Th9, Th17, Th22, T follicular helper, and T regulatory cells under unique cytokine-polarized milieus (Figure 1). These subsets of Th cells secrete diverse effector molecules contributing to cell-mediated or humoral immune responses, inflammation or immunoregulation. CD8^+^ T cells release a variety of cytotoxic molecules such as perforins, granzymes, and IFN-γ, which kill the target cell, e.g., host cells infected with viruses or intracellular bacteria. Once the infection is resolved, antigen-specific CD4^+^ and CD8^+^ effector T cells decline in number and a small population is usually maintained as antigen-specific memory CD4^+^ and CD8^+^ T cells.

#### 3.2.3. Innate Immunity

Traditionally the evaluation of vaccine efficacy has been based on measuring of specific antibody and T cell readouts or challenge experiments in animal models. However, innate immune activation, which has an important impact on the final outcome of vaccine efficacy and safety, is rarely measured. Early innate immune responses have a well-established role on the eventual down-stream adaptive immunity as well as on early inflammation with potential unwanted side effects of vaccines. It is therefore expected that innate responses may represent a correlate of vaccine efficacy. For many vaccines, the traditional outcome of a vaccination is assessed several weeks after vaccination, however determination of early correlates of vaccine efficacy could speed-up the rational design of vaccines. A well-known problem in evaluating vaccine responses is the large variation in immune responses of the host which is seen in both human and veterinary species. This is driven by heterogeneity in age, genetics, and environmental factors including previous infections, stress or changes in microbiome following antibiotic treatment [58,59,60]. This variation is also a problem in challenge experiments performed for veterinary vaccines and results in poor statistical power of many vaccine trials.

Systems vaccinology provides a solution to these issues. This approach employs multiplexed immune profiling technologies combined with computational modelling to evaluate vaccine responses. This can be applied to peripheral blood samples very early after vaccination, providing molecular signatures of protective immune responses [61]. In fact, it was demonstrated that transcriptomic data can be most informative if analyzed using blood transcriptional modules (BTM) that were created on the basis of highly interacting genes [62,63]. In recent years this approach has continuously been applied to many human studies and, as new technologies have emerged, refined by integration of data from a number of “omics” technologies [64]. In general terms, the data generated using BTM provide information on changes in immune cell population distribution, cellular processes, such as cell cycle and transcription, leukocyte-specific signaling pathways, leukocyte migration, activation of particular immune cell types such as dendritic cells and T cells, inflammation, coagulation, platelet activation, antiviral responses, antigen presentation, immunoglobulin production, or on metabolic processes relevant for immune responses [60,63,65].

Recently such methods have also successfully been adapted to veterinary species including sheep and pigs [66,67] and demonstrated their power to detect and explain immunological processes occurring in tissues such as the injection site of a vaccine using peripheral blood as source. These data can predict vaccine responses and enable a detailed characterization of immune responses induced by different formulations [67]. Interestingly, the correlation patterns of BTM with adaptive responses were seen across multiple species.

In summary, systems vaccinology pipelines can be used to dissect the impact of vaccine components and their formulations on the immune system and thereby help to identify improved delivery systems and immunostimulants. It is possible to identify innate correlates and biomarkers of suitable vaccines to improve formulations, select optimal immunostimulants and compare different batches of vaccines. Finally, such analyses have been used to identify pathways responsible for the heterogeneity in vaccine responses caused by age, nutrition, stress, genetics and the microbiome (reviewed in [64,68]).

## 4. Choice of Vaccine Antigen

### 4.1. Rational Antigens for Antibody Immunity

Should immunological data indicate that a disease can be prevented by an antibody response, it is important to select an appropriate antibody-inducing antigen. Induction of robust antibody responses by vaccines requires inclusion of relevant antigenic epitopes for B cells as well as T cell peptide epitopes to elicit appropriate T helper (Th) cell activation and assistance to B cells. Hence, selection of the optimal antigen is imperative in vaccine design.

A successful strategy in the selection of the antigenic determinants as “subunit” vaccines is the isolation and inactivation of essential components of the pathogenic organism [69]. Chemically inactivated toxins isolated from bacteria have been exploited for diphtheria and tetanus vaccines. This approach has also been pursued for subunit vaccines based on purified polysaccharides of Gram-negative bacteria, such as those developed for *Streptococcus pneumoniae*, *Haemophilus influenzae* or *Salmonella typhi Vi*. Saccharide-based vaccines have also been developed in multivalent forms to provide protection against numerous serotypes and serogroups [70].

Current strategies utilize reverse vaccinology approaches to select promising glyco- or protein-derived antigens based on structural studies of the target epitopes of potent antibodies [71,72]. Rational, structure-guided vaccine design can also involve the development of structurally simpler immunogens that present well-defined minimal epitopes targeted by neutralizing antibodies, serving as epitope mimics for elicitation of more focused immune responses [73]. When refined recombinant or synthetic subunit vaccines are designed, the structural features of the antigen may strongly impact the desired antibody-response.

Structural antigen vaccinology is a structural biology approach to design immunogenic antigens. It rationally aims to generate an effective antibody-inducing vaccine antigen, combining experimental methods such as X-ray crystallography, nuclear magnetic resonance (NMR), molecular biology, electron microscopy and mass spectrometry, with computational methods including molecular modeling, virtual screening and epitope prediction [74]. The identification of an antigen candidate is typically based on its cellular location. Although computational approaches exist to predict the protein localization in the cell, a leading technique for antigen identification is mass spectrometry, which allows the assessment of surface structures. Knowing the structure of an antigen will provide important insights into the tertiary structure and position of the potential epitopes. Moreover, structures of antigen-antibody complexes enable the elucidation of the molecular nature of host-pathogen interactions and of pathogen- or vaccine-induced antibody responses. The first step of this approach is the three-dimensional structure determination of the antigen using structural biology tools such as X-ray crystallography, cryo-electron microscopy and NMR. Epitope mapping of an antigen that is recognized and bound by antibodies is key to vaccine development This will provide a comprehensive dataset at atomic level which is necessary to engineer new constructs with better properties in terms of elicitation of the antibody response, stability in solution and ease of production.

The aim of this re-engineering process is to obtain an antigen that is more effective in eliciting an optimal B cell response. Based on the analysis of antigen-antibody interaction network, the candidate vaccine antigen may contain only those residues essential to reproduce the protective epitopes. Molecular modeling is extremely helpful in designing optimal re-engineered antigens, through the identification of mutations that stabilize immunogenic conformations of epitopes. Moreover, residues outside the epitope can be mutated to improve antigen production yields. Another important issue in antigen re-engineering is the improvement of antigen thermal stability, through the identification of stability-enhancing mutations or through the insertion of the designed antigen in stable protein scaffolds. Eventually, the immunogenicity and efficacy of the candidate vaccine must be tested in animal models [75].

While glyco- or peptide-based subunit vaccines offer improved safety and more precise targeting, they usually require conjugation to immunogenic molecules (i.e., carrier proteins or immunogenic Th sequences), or presentation in multimeric format (virus-like particles or nanoparticles) to achieve optimal immune responses [76], which can be further enhanced by inclusion of an immunomodulatory adjuvant. Notably, issues of immunodominance are a critical aspect to designing optimized vaccines and need to be considered when selecting the epitope in order to elicit focused, protective broadly neutralizing antibody responses directed against naturally non-immunodominant conserved epitopes [77].

Another key element for rational vaccine design is the presentation of the antigen in its relevant native-like conformation, enabling optimal antibody recognition of the antigenic epitope within the quaternary protein structure. Several approaches have been developed to modulate these epitopes to favor conformationally relevant states of the antigen [78], including side chain cross-linking, grafting the epitope into a larger scaffold and other rationally designed chemical modifications leading to enhanced antibody binding affinity [79]. With the development of chemical technologies, such as covalent conjugation of immunogenic carrier proteins to polysaccharides, subunit vaccines have substantially advanced and have provided improved memory responses, thus addressing a key limitation suffered from saccharides vaccines. [80].

Several prototypes of structure-based vaccine development have been evaluated. One example represents the vaccine development against respiratory syncytial virus (RSV). In this case, specific epitopes belonging to the F glycoprotein (sites 0, III, and V) present potent neutralizing activity, 10 to 100 times greater than that observed for clinically used monoclonal antibody palivizumab (Synagis^®^). However, these key epitopes are well exposed only in the pre-fusion trimeric conformation of the RSV-F protein. Structural vaccinology was fundamental to generate stabilized pre-F trimers that preserved key epitopes in the proper conformation to elicit the most neutralizing activity in human sera [81,82]. Another example is the licensed 4-component vaccine against *N. meningitidis* serogroup B (MenB) composed of three recombinant proteins and a bacterial membrane vesicle selected using a reverse vaccinology approach (Section 2.2) [13]. Structure-based design was subsequently used to generate a vaccine made of chimeric antigens, by retaining epitopes from two antigens (fHbp and PorA), thus potentiating its power to elicit functional humoral immune responses against MenB [83]. These two examples illustrate that structural vaccinology can generate novel vaccine antigen candidates with improved characteristics for antibody-based strategies.

The potential improvement in the identification of antigenic determinants expressed on cancer cells enabled the design of therapeutic subunit vaccine to treat cancer [84,85,86]. Although the use of cancer vaccines awaits meaningful clinical benefits several tumor-associated carbohydrate antigens (TACAs) are currently largely validated and have been used to design promising molecular vaccines. Conjugation of these TACAs to proteins or the development of altered-self, i.e., more immunogenic, TACA analogues are examples of current strategies to make TACA-based vaccines able to break immune tolerance and elicit cancer antigen-specific antibodies [87].

### 4.2. Rational Antigens for Cell-Mediated Immunity

**Figure 2:**
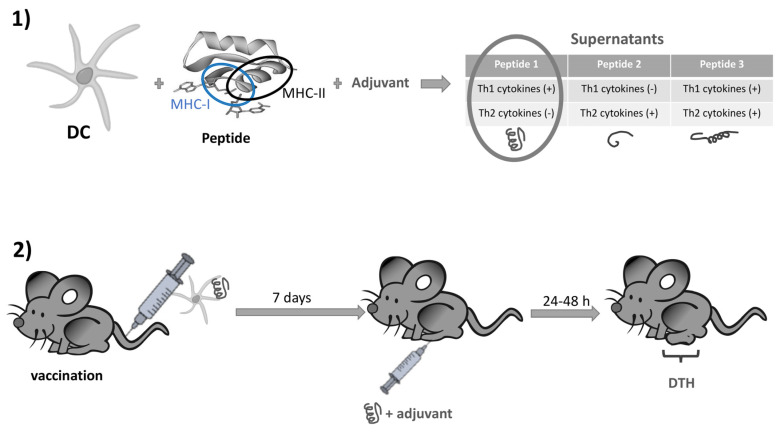
Methodology using dendritic cells (DCs) and adjuvants to select vaccine antigens for cell- mediated immunity. (1) DC loaded with peptides, good Major histocompatibility (MHC)-I and MHC-II binders and a Th1 adjuvant are checked for Th1 and Th2 cytokine releases. (2) Peptides inducing Th1 cytokines are selected and validated in mice vaccinated with DCs loaded with the peptides and inoculated into the footpads to measured delayed hypersensitivity (DTH) reactions. Peptides inducing strong DTH reactions are considered good candidates for future vaccines.

A considerable challenge in vaccinology is the design of vaccines against pathogens for which antibody immune responses are not protective [88]. To develop T cell vaccines, it is important to have an antigen that elicits potent cellular immune responses as well as an easy methodology to test the validation of the vaccine design in vivo and in vitro. In this regard, proteomics approaches with virulence factors of pathogens that induce potent CD4^+^ and CD8^+^ T cell responses are worthy methods to de-sign vaccines using reverse vaccinology approaches [89]. Using *Listeria monocytogenes* as a model pathogen Kono and co-workers and Calderon-Gonzalez and co-workers and DCs loaded with peptides of two virulence factors of this pathogen, listeriolysin O (LLO) and the glyceraldehyde-3-phosphate dehydrogenase (GAPDH) prepared vaccines that confer listeriosis protection [90,91]. Later, a methodology that combines bioinformatic analysis to screen for the best MHC binders, delayed type hypersensitivity (DTH) to test the best T cell mediated inducers and analysis of cytokines released by DC loaded with the epitopes and an adjuvant to search for epitopes inducing only Th1 and not Th2 cytokines [92] (Figure 2). Consequently, MHC binding epitopes and inducers of Th1 cytokines were selected and included in the peptide sequences contributing to immune protection. DCs loaded with those epitopes that meet the highest MHC binding, T cell induction and high levels of Th1 cytokines were validated as efficient epitopes for vaccination against *L. monocytogenes* challenge. This approach helps to predict other epitopes for vaccines against listeriosis and other pathogens as mycobacteria and streptococci [93]. Other proteomics approaches similar to this one also helped to design.

## 5. Choice of Immunomodulation 

### 5.1. Rational Vaccine Adjuvant Design

For the past few decades, the focus of new vaccine development has been on the antigen(s) and new ways to present antigen. Optimization of the immune response with the use of different types of adjuvant has received less attention. Typically, when preliminary studies with a “standard” adjuvant does not show the desired (protective) response, the researchers will seek another type of antigen, instead of searching for an appropriate adjuvant that may provide the suitable or preferred immune response. Often the desired adjuvant or formulation is unknown or not available. Nevertheless, there have been noteworthy achievements in improving existing vaccines by introducing specific adjuvants as well as new vaccine delivery methods [1,94].

Live attenuated vaccines, however, do not usually require adjuvants since the resulting immune response is a result from the attenuated microbe and its attempt to multiply. However, adjuvants come in many forms [95] and may have many functions as shown in Table 1.

### 5.2. Rationally Designed Vaccine Adjuvants

Molecularly defined subunit vaccines are generally safer but often lack immunogenicity, due to the absence of key additional structural components needed for efficient activation of innate immunity. Such vaccines require the coadministration of an adjuvant [96]. Few adjuvants are already licensed as part of vaccine formulations and some others are being used in the clinic [97]. However, the development of adjuvants has been traditionally an empirical process [98] as their molecular mechanisms of adjuvant activity are not fully understood [95,99,100], hindering the rational design of improved, less toxic adjuvants for optimal matching with selected vaccine antigens [101]. For long the immunological function of vaccine adjuvants has been subject of speculation [95].

It is not straightforward to translate the newly discovered mechanisms of adjuvant activity to generally applicable approaches for rationally designed vaccines. Thus, a thorough knowledge of adjuvants and their immunological effects is needed to realize the full potential of rational vaccine design. [102]. These critical vaccine elements come in many forms and serve to initiate, accelerate, amplify, improve and prolong the immunological responses to antigens. Vaccine adjuvants have been classified in vaccine antigen delivery systems (facilitating immune signal 1) or director immunostimulatory molecules (facilitation immune signal 2) or a combination of both [95].

The most common and long-time used immunoadjuvant alum has been extensively reviewed before [100,101], but mostly induces improved antibody responses. Other more recent clinical stage adjuvants include oil-based emulsions, such as MF-59, saponins, ISCOMs, oxoadenines, C-type lectin ligands. One prominent category includes Toll-like receptor agonists such as poly(I:C), the family of imidazoquinoline adjuvants (resiquimod, imiquimod), GLA, CpG motifs or flagellin. Another adjuvant group includes particle adjuvants, mimicking micro-organism structures, including nano- or microparticles or virus-mimicking polymers, such as polyphosphazene polyplexes PCPP and PCEP. Modularly composed AS0 adjuvants of Glaxo Smith Kline (GSK) and the CAF adjuvants of the Statens Serum Institute, have been extensively reviewed before [103,104]. All these systems will not be described in this review. Instead, a limited number of prototype examples of rationally designed vaccine adjuvants are described below.

#### 5.2.1. Natural Lipid A as Source of Inspiration for New Adjuvants

The lipid A molecule is the immunostimulant component of Gram-negative bacterial lipopolysaccharide (LPS). It consists of a β-(1→6)-linked diglucosamine backbone with different patterns of acylation and phosphorylation [105]. The lipid A structure activates the TLR4/MD-2 immune receptor complex that mediates gene expression and secretion of pro-inflammatory cytokines. Through activation and regulation of various dendritic cell functions TLR4 activation bridges innate and adaptive immunity by inducing signals that are vitally involved in the initiation of adaptive immune responses [106]. TLR4 activation by lipid A is structure dependent. The bis-phosphorylated hexa-acylated lipid A species with a 4/2 distribution of the acyl chains (*E. coli* type) is the most toxic and its recognition and binding by the TLR4/MD-2 binary complex can lead to sepsis. Changing the number and length of the acyl chains or the phosphate decorations decreases its recognition by the immune system receptor [107,108]. A vast diversity of lipid A molecules can be found as moieties of Gram-negative bacterial LPS. Acyl chains can be absent (de-acylation) or hydroxylated [109] or more acyl chains can be added such as palmitate limiting immune system recognition [110]. The removal of the anomeric phosphate group of the *E. coli* type lipid A makes the molecule only moderately active. The phosphate groups can be absent or replaced by monosaccharides [111,112,113,114] or can also be decorated by phosphoethanolamine or cationic monosaccharides as 4-amino-4-deoxy-β-l-arabinose to neutralize their negative charge altering immune recognition [115,116]. In addition, in nature, the glucosamine disaccharide can be replaced by the 2,3-diamino-2,3-dideoxy-d-Glc disaccharide [117,118]. There is a vast number of lipid A molecules that are partial agonists and antagonists of TLR4. Particularly, gut microbiota have proven to be a tremendous library of lipid A molecules with different activities [119]. Lipid A is also capable of activating C-type lectin receptors (CTLR) [120], and caspases [121,122,123,124]. In addition, some atypical LPSs can activate TLR-2, although the chemical determinants responsible of this interaction are not yet clear [113,125].

**Figure 3:**
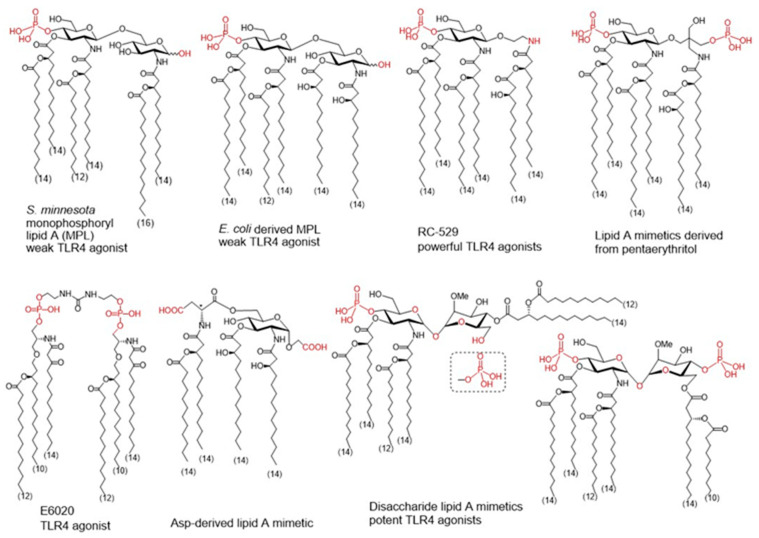
Monophpsphoryl lipids (MPLs)Ls and synthetic lipid A mimetics as promising vaccine adjuvant candidates.

For the structural reasons explained above, some natural LPS could be used as vaccine adjuvants. For example, *Brucella abortus* LPS showed promising results in various formulations [126,127], as well as the naturally occurring monophosphorylated lipid A of *Bacteroides thetaiotaomicron* and *Prevotella intermedia* [128]. There are also LPS-modified adjuvants like the *O*-deacylated lipooligosaccharide from *E. coli* J5 [129] or the broadly used adjuvant Mono-Phosphoryl Lipid A (MPL) (Figure 3) [130]. MPL is derived by the removal of the anomeric phosphate group of the hepta-acylated lipid A of *S. minnesota* R595, which reduces significantly the TLR4-driven activity and toxicity. Decreased TLR4 activation induced by MPL derives from a less efficient dimerization of TLR4/MD-2/MPL complexes due to the absence of the phosphate group which the weakens proteins’ interaction at the dimerization interface [131,132,133,134]. MPL formulations are incorporated in approved vaccine preparations; moreover, MPL has also been used as a starting point of further modifications to develop new adjuvants [135,136].

To improve specific adjuvant properties and to modify or enhance immune stimulating activity, different synthetic lipid A analogues and lipid A mimetics have been prepared as vaccine adjuvant candidates. Chemical synthesis provides access to structurally defined molecules free of any biological contaminations and allows tremendous improvements in structure-activity relationships studies. Basically, all synthetic lipid A mimetics mirrored the basic architecture of their parent lipid A and were composed of a hydrophilic polar/charged head group and a hydrophobic lipid region. Simplification of the lipid A structure by replacing one or both glucosamine (GlcN) rings for linear or branched aglycons. Thus, GSK Biologicals developed aminoalkyl glucosaminide phosphates (AGPs) where the proximal GlcN ring of lipid A was omitted furnishing in this way a polar head group with rationalized structure [137]. Variable β-hydroxyacyl and alkyl chains were chemically attached to the polar head group and selected AGPs reached pre-clinical/clinical development which highlighted RC-529 as a potent vaccine adjuvant (Figure 3) [138]. Lipid A mimetics containing pentaerythritol in place of proximal GlcN residue were developed by Biomira Inc. (Edmonton, AB, Canada) as potent cytokine secreting agonists. A pentaerythritol-derived lipid A mimetic demonstrated adjuvant properties through enhancement of antigen-specific T cell activation in a synthetic liposomal vaccine system [139]. Replacement of the distal GlcN moiety of lipid A for the acidic amino acid Asp and the glycosidic phosphate group for a carboxyl group (Asp-derived lipid A mimetic) ensured immunostimulating potential despite of only four lipid chains attached [140]. Further structure simplification led to development of an acyclic lipid A mimetic E6020 (Eisai, Tokyo, Japan) consisting of a hexaacylated flexible linear backbone (Figure 3) [141]. The antibody response to vaccines co-administered with this TLR4 agonist E6020 led to a mixed Th1/Th2 response [142]. In addition, newly developed disaccharide lipid A mimetics profited from a conformational rigidity of their nonreducing disaccharide backbone and exhibited picomolar affinity for TLR4 [143]. Thus, adjustable TLR4 activation and graded induction of cellular pro-inflammatory responses renders these glycolipids promising vaccine adjuvant candidates [144].

#### 5.2.2. Rational Design of Allostatine Adjuvant

Alloferons are a group of natural peptides isolated from insects that can stimulate human natural killer (NK)cell cytotoxicity towards cancer cells. These peptides are originally isolated from the hemolymph of maggots from the blowfly *Calliphora vicina*. A striking feature of larval hemolymph is that the hemocytes possess a cytotoxic activity functionally analogous to human NK cells. When stimulated by bacteria, these larvae produce high levels of potent defensive molecules typical of the insect immune system which accumulate in the hemolymph [145]. These include Alloferon1 and Alloferon2, two virtually indistinguishable peptides consisting of 13 and 12 amino acids respectively. Both Alloferons stimulate in vitro natural cytotoxicity of human blood mononuclear cells. Alloferon1 stimulates natural cytotoxicity in human in vitro models as well as antiviral and anticancer activities in mouse models in vivo. In a search for a molecule with higher antitumoral activity and cancer immunotherapy potential, the primary structure of Alloferon1 has been modified to obtain the peptide Allostatine, by changing two amino acids. The substitution of two amino acids in the Alloferon sequence was designed to simulate a pattern typical of the human immunoglobulin [146] belonging to the immunoglobulin heavy chain CDR3 region which is very well conserved between mammalian genomes, notwithstanding that CDR3 region is the most variable section of immunoglobulins. The molecular mechanism of action of Allostatine is still not clear. In silico simulations suggested NKG2D as a possible target for this peptide, while in vitro experiments showed that low, ng/mL concentrations of Allostatine in culture medium cause rearrangement of NK and T cells receptors, stimulating NK cells cytotoxic activity against cancer cells increasing the number of IFN-gamma and IL2 producing cells [147]. Allostatine manifested strong adjuvant properties in a mouse P388/DBA2 tumor transplantation model when combined with a vaccine consisting of X-ray inactivated tumor cells. While the vaccine alone demonstrated only a weak tumoristatic effect in about 25% of recipients, The vaccine in combination with Allostatine caused a tumoristatic effect in approximately 65% of recipients and prevented tumor occurrence in another 30% (resulting in a positive influence on 95% of recipients) [146]. Hence, the designed peptide Allostatine therefore, possesses characteristics of an adjuvant boosting cancer therapeutic vaccines efficacy.

#### 5.2.3. Nod2 Agonists as Rationally Designed Vaccine Adjuvants

Nucleotide-binding oligomerization domain-containing protein 2 (NOD2) is a cytoplasmic pattern recognition receptor involved in both innate as well as adaptive immune responses and therefore constitutes an excellent target for rationally designed vaccine adjuvant ligands [148]. Muramyl dipeptide (MDP) is the smallest structural subunit of bacterial peptidoglycan capable of eliciting NOD2 activation, which leads to pro-inflammatory and antimicrobial responses characterized by the secretion of cytokines, induction of autophagy and production of antimicrobial peptides [148]. Activation of NOD2 itself is sufficient to shape the adaptive immune response towards a Th2 response [149]. Incidentally, NOD2 agonistic activities have been shown to strongly correlate with their adjuvant properties [150]. NOD2 agonists also amplify the adjuvant potential of TLR ligands and alter the magnitude, persistence and the type of response towards the Th1 type [151]. Interestingly, engagement of NOD2 proved to be essential for antigen-specific mucosal and systemic responses of mucosal vaccines [152,153]. This is noteworthy in light of the fact that the majority of pathogens gain entry through mucosal sites and given the shortage of mucosal adjuvants, the use of NOD2 agonists in cancer vaccines was also highlighted [154].

**Figure 4:**
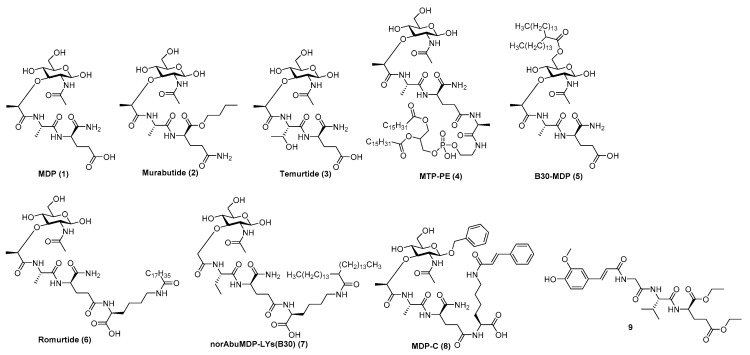
MDP (**1**), its most prominent pharmacologically active derivatives (**2**–**8**) and a desmuramylpeptide derivative with NOD2 activity (**9**).

Although MDP (Figure 4 (**1**)) is predominantly responsible for the efficacy of Freund’s complete adjuvant, it suffers from pyrogenicity and rapid elimination as a single administrated molecule [151]. To that end, many chemically modified derivatives of the parent MDP molecule (Figure 4) have been synthesized with the aim of reducing its toxicity and improve its pharmacokinetic properties. Of the hydrophilic derivatives known to mainly induce a Th2-type response, murabutide (Figure 4 (**2**) and temurtide (Figure 4 (**3**) emerged as the most interesting candidates for further development as vaccine adjuvants, while muramyl tripeptide phosphatidylethanolamine (mifamurtide; MTP-PE) (Figure 4 (**4**), B30-MDP (Figure 4 (**5**) and romurtide (MDP-Lys (L18) (Figure 4(**6**) were the most prominent lipophilic derivatives, which tend to augment the Th1-type immune reaction [154].

Substitution of the *N*-acetyl group by an *N*-glycolyl group produces *N*-glycolyl-MDP, a distinctive feature of the BCG vaccine, which exhibits superior adjuvant activity. The l-Ala position is also available to other amino acids, since l-Ser, l-Val and l-Thr peptide analogs retained the adjuvant activity of the parent molecule [150]. The d-iGln moiety, on the other hand, is less amenable to substitution; it can only be replaced by either d-Gln or d-Glu (including their esterified forms) as exemplified by murabutide or muradimetide [148]. Murabutide is apyrogenic and well tolerated by humans [155], therefore its adjuvant effect was assessed following administration with the fluid phase of tetanus toxoid vaccine, in which case significantly higher IgG levels to toxoid were found in the group receiving vaccine with murabutide compared to the group given the vaccine alone [156]. Its adjuvant capacities were further underlined using a combination of murabutide and a synthetic hepatitis B antigen with increased levels of antigen-specific antibodies [157]. Recently, Jackson et al. reported on the ability of murabutide to induce a robust and durable IgG and IgA antibody response to Norwalk virus following intranasal vaccination which proved its ability to act as a potent mucosal adjuvant [153]. Temurtide is a threonine-based MDP derivative, used as an active principle of the SAF-1 formulation, which has been tested as adjuvant in preclinical trials in guinea pigs and mice. It successfully increased the formation of IgG2a antibodies against HBsAg [158].

The introduction of lipophilic groups into the structures of NOD2 agonists has been shown to strongly enhance the cellular immune response and overall increased the immunostimulatory adjuvant activity of the compounds. MTP-PE (Figure 4(**4**) was evaluated in Phase I clinical trials as a vaccine adjuvant in human immunodeficiency virus type I vaccines, but failed to improve their immunogenicity, while causing increased reactogenicity [159]. Decoration of the muramyl residue 6-OH group with a lipophilic linear/branched fatty acid structural feature resulted in derivatives with noteworthy adjuvant properties, as exemplified by B30-MDP [148]. Further structural optimization, which entailed the extension of the peptide stem with *N*-stearoyl-l-Lys, led to the discovery of MDP-Lys(L18) (also known as romurtide or muroctasine). A combination of MDP-Lys(L18) and B30-MDP has shown promising results in mice, which produced higher antibody titres against rHBsAg after intraperitoneal injection [160] and increased the humoral and cellular response against an inactivated hantavirus vaccine [161]. A close structural analog norAbuMDP-Lys(B30) (Figure 4 (**7**), which proved effective as an adjuvant for *Borrelia burgdorferi* antigen rOspA, also features a B30-acylated Lys-extension [162]. Similarly, MDP-C (Figure 4 (**8**) carries a *N*-cinnamoyl-l-Lys moiety and showed promising results in a mouse model by increasing the levels of anti-HBs antibodies [163]. Desmuramylpeptides are MDP derivatives in which the sugar *N*-acetylmuramyl moiety (Mur*N*Ac) is replaced by a hydrophobic group. The *Trans*-feruloyl moiety has recently been identified as an excellent Mur*N*Ac mimetic, resulting in a low nanomolar NOD2 agonist (Figure 4 (**9**), which induced ovalbumin-specific IgG titers in a mouse model of adjuvancy [164].

#### 5.2.4. QS-21-Based Synthetic Saponin Adjuvants

**Figure 5:**
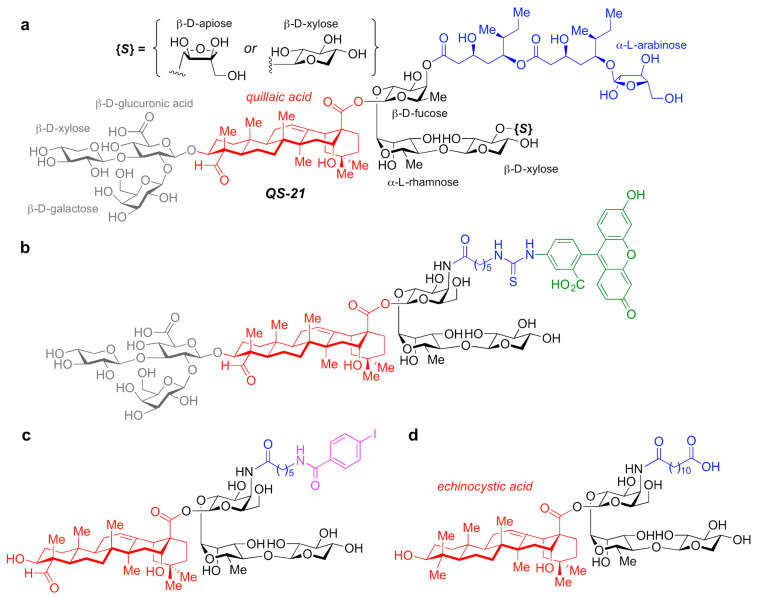
(**a**) Chemical structure of QS-21 saponin natural product adjuvant. (**b**–**d**) Synthetic saponin variants based on QS-21.

A number of adjuvant analogues have been developed based on natural product adjuvants leveraging detailed structure–activity relationship studies, such as synthetic saponin variants derived from QS-21 (Figure 5a) and the *Quillaja* saponin (QS) family [165].

In particular, QS-21 is a saponin natural product with a long history and great potential as an adjuvant. It elicits both antibody and cellular immune responses, including cytotoxic T lymphocytes, and has been recently approved in combination with MPLA as part of the AS01 adjuvant system in vaccines against malaria and shingles [166]. However, despite its promise, QS-21 suffers from several drawbacks, including scarcity, heterogeneity, chemical instability and dose-limiting toxicity, which have hampered its more widespread use in human vaccines. As such, the discovery of new, improved QS-21 variants has been at the forefront. In this context, Fernández-Tejada et al. identified key structural features of QS-21 that are important for adjuvant activity [167,168] and have developed a variety of simplified, synthetically accessible saponin derivatives that induce antibody responses comparable to QS-21 with drastically reduced toxicity (Figure 5b–d) [169,170]. Moreover, these structurally simpler saponin scaffolds were leveraged for the development of saponin variants bearing fluorescent and radioiodinated tags (Figure 5b,c). These saponin probes were exploited in imaging and biodistribution studies that revealed internalization of active variants into dendritic cells and accumulation in the lymph nodes, which suggests a role for adjuvant-active QS variants in the trafficking of antigens by APCs to the draining lymph nodes [169].

Additional studies on the detailed immunological profile of these saponins are warranted and in progress, with the aim to elucidate the precise functional and molecular roles of these adjuvants, also in the context of adjuvant systems and anti-cancer vaccines.

### 5.3. The VLP-Based Vaccine Platform and CpG ODNs as Immunoprotective Vaccine Adjuvants

The spread of COVID-19 highlighted the need for swift vaccine development in a global pandemic and over 320 vaccine trials were conducted worldwide at time of writing [171,172]. Historically live attenuated viral vaccines could be selected as one of the most effective and protective platforms during a pandemic. However, the challenge involved in rapid generation of an attenuated SARS-CoV-2, as well as recent advances in molecular biology and immunology, promoted the evaluation of faster alternative strategies, such as Virus Like Particles (VLPs), as a convenient and effective approaches against controlling COVID-19 pandemic.

VLPs are macromolecular self-assembling structures that closely resemble the native forms of viruses. One of the superior features of these “stunt viruses” is that they are non-infectious since they lack viral genetic content. Therefore, VLPs are safer than whole-pathogen-based vaccines such as those containing attenuated viruses. VLPs can be developed through expression of individual viral structural proteins following transfection which can self-assemble into the VLPs before being released into the extracellular environment from the producer cells. The success and licensing of the multivalent VLP-based vaccines for human papilloma virus and Hepatitis B validated the safety and efficacy of the vaccine concept for VLPs [173,174], which supports the utilization of this platform to further develop effective vaccines against newly emerging infectious agents. A Coronovirus-like particle VLP vaccine is being evaluated in Phase 2/3 trials [175].

In the case of SARS-CoV2 for example, four structural proteins of SARS-CoV-2, namely, spike, envelope, membrane and nucleocapsid could be cloned within proper expression vector(s) and expressed in a suitable producer cell line that could range from mammalian to insect, to even yeast or plant cells. The immunogenicity and strength of protective capacity of these vaccine candidates could be further amplified by the use of a proper Th1 immunity-supporting biological adjuvants such as CpG oligodeoxynucleotides (CpG ODN hereafter) [176]. Development of effective vaccine mediated immune responses relies on the use of vaccine adjuvants capable of enhancing and directing the adaptive immune response to the antigen. When used as vaccine adjuvants, type I interferon inducing agents can elicit both potent effector/memory T cell responses and humoral immunity.

Specific sequences of single stranded synthetic oligodeoxynucleotides containing unmethylated cytosine-phosphate-guanine oligodeoxynucleotide motifs (CpG ODN) stimulate type I interferon production via TLR9. Based on their differential activation of immune cells, four major classes of synthetic CpG ODNs have been defined. The K class ODNs are potent B cell activators that stimulate TNF-α secretion not interferon-α (IFNα), while D, C-, and P-class ODNs induce variable amounts of IFNα secretion [177,178]. The D-class ODNs are the most potent IFNα inducers but form multimers which complicates their GMP manufacture. There have been only three clinical trials to date evaluating D-class ODNs as either a vaccine adjuvants and/or immunotherapeutic applications. All three studies harnessed a stabilized version of this ODN class following packaging into virus like particles consisting of the bacteriophage Qß coat protein. Interestingly, our recent studies confirmed that inclusion of CpG ODNs within SARS-CoV-2 VLPs elicited pronounced humoral and cell mediated immunity against COVID-19 infection (Gursel et al., 2021 unpublished data).

## 6. Outlook/Closing Remarks

The complexity of the natural immune response and the potential immune escape mechanism(s) of particular pathogens complicate the design and its predictive value of a successful vaccine and the selection of critical vaccine elements. The vaccine components described above are very useful tools to design considerably different vaccine concepts that are necessary to evoke the desired immunological correlates of protection and, if necessary, to outsmart the pathogen by artificially induced immune responses. In times of emerging infections, the speed of efficient vaccine development becomes a critical factor to prevent health threatening disease. Hence, having a detailed knowledge and the availability of critical vaccine components is imperative to the prevention of future life-threatening diseases.

Specifically, versatile vaccines, which consist of elements that are readily available, to rapidly produce the antigens expected to provide successful immune targets are highly needed in emergency situations. Therefore, vaccines based on genetic approaches, such as RNA- and DNA vaccines, or recombinant vector vaccines, or synthetic peptide vaccines are very useful tools which can be engaged rapidly as soon as the required genetic sequence of the presumed target antigen becomes available. Mathematical modeling and deep learning artificial intelligence (AI) applications involving the prediction of viral mutation(s) may undoubtedly help to anticipate the next generation of viral disease. For example, with RNA sequences of one generation of a virus acting as inputs, and the RNA sequences of the next generation acting as outputs, an algorithm was developed to predict RNA sequences of successive generations [179]. In addition, readily available prototype vaccine adjuvant systems which have proven to evoke, amplify and accelerate particular types of immune responses that are expected to provide protection will be useful to obtain a workable vaccine. When the immunological correlate of protection for the emerging disease remains undefined readily available prototype adjuvant systems may be employed for inclusion in experimental vaccine prototypes. Yet, the global availability of sufficient quantities of these elements may become another critical factor in times of urgent mass vaccination efforts.

## Figures and Tables

**Table 1:** Pharmaceutical and Immunological Functions of vaccine adjuvants.

*Pharmaceutical function (affecting vaccine antigen delivery)*
Prolong antigen residence time at the site of administration
Protect the nature of the antigen
Prevent the antigen from degradation (improve stability)
Protect the 3D structure of the exposed antigen epitope(s)
Induce an environment that mimics the infection
Secure absorption into the lymphatics over systemic circulation
Decrease the number of boosters required for successful immune response
Have a good safety and toxicological profile
*Immunological function (impact on immune function)*
Attract antigen presenting cells to the site of administration
Augment immune response type: e.g., Th1, Th2, Th3 or Th17
Augment the generation of memory cells
Augment mucosal, or systemic responses
Improve the generation of neutralizing antibodies and /or effector T cells

## Data Availability

No new data were created or analyzed in this review. Data sharing is not applicable to this article.
